# Papillary Traction Due to the Posterior Form of a Bilateral Persistent Fetal Vasculature Mimicking Papilledema

**DOI:** 10.7759/cureus.58738

**Published:** 2024-04-22

**Authors:** Hamza Lazaar, Taha Boutaj, Meryem Sefrioui, Boutayna Azarkan, Saad Benchekroun, Noureddine Boutimzine, Abdellah Amazouzi, Lalla Ouafa Cherkaoui

**Affiliations:** 1 Ophtalmology, Hopital des Specialités, Rabat, MAR; 2 Ophthalmology, Hopital des Specialités, Rabat, MAR

**Keywords:** fundus examination, persistent hyperplastic primary vitreous, traction, pseudopapilledema, persistent fetal vasculature

## Abstract

Persistent fetal vasculature (PFV), or persistent hyperplastic primary vitreous (PHPV), is a congenital developmental disorder characterized by a failure of resorption of the hyaloid system. It typically presents unilaterally and has three forms: anterior, posterior, and mixed. In this case report, a seven-year-old patient, without specific personal or family medical history, was referred from the pediatric department for bilateral papilledema. The patient had a best-corrected visual acuity of 20/20 (Logarithmic Measure of Angle of Resolution (LogMAR): 0) in both eyes. Fundus examination of both eyes revealed congested pseudopapilledema with a short, mobile, brownish band extending from the optic disc towards the vitreous cavity. Ocular ultrasound of both eyes showed a fine hyperechoic line pulling on the optic nerve head, and papillary optical coherence tomography (OCT) showed a papillary traction syndrome. The diagnosis of a posterior and bilateral form of persistent fetal vasculature with papillary traction was established.

## Introduction

Persistent fetal vasculature (PFV), or persistent hyperplastic primary vitreous (PHPV), is a congenital developmental disorder marked by a failure of resorption of the hyaloid system [1]. It typically presents unilaterally, rarely bilaterally, and comes in three distinct forms: an anterior form, a posterior form (the rarest), and a mixed form [2]. It is considered one of the main differential diagnoses of retinoblastoma [2]. Also, it can cause serious complications such as angle-closure glaucoma, hyphemia, vitreous hemorrhage, and tractional retinal detachment [3]. We report an extremely rare case of a posterior and bilateral form complicated by papillary traction mimicking bilateral papilledema.

## Case presentation

We report the case of a seven-year-old girl, without notable personal or family medical history, referred from the pediatric department in our institution for further management of bilateral papillary edema. She had previously undergone a brain MRI, which revealed no abnormalities.

Upon ophthalmological evaluation, the patient had a best-corrected visual acuity of 20/20 (Logarithmic Measure of Angle of Resolution (LogMAR): 0) in both eyes. Pupils were equal and reactive, with no leukocoria or strabismus. The anterior segment examination of both eyes showed clear corneas with normal diameter, well-depth anterior chambers, no aniridia, and clear lenses without opacities.

**Figure 1:**
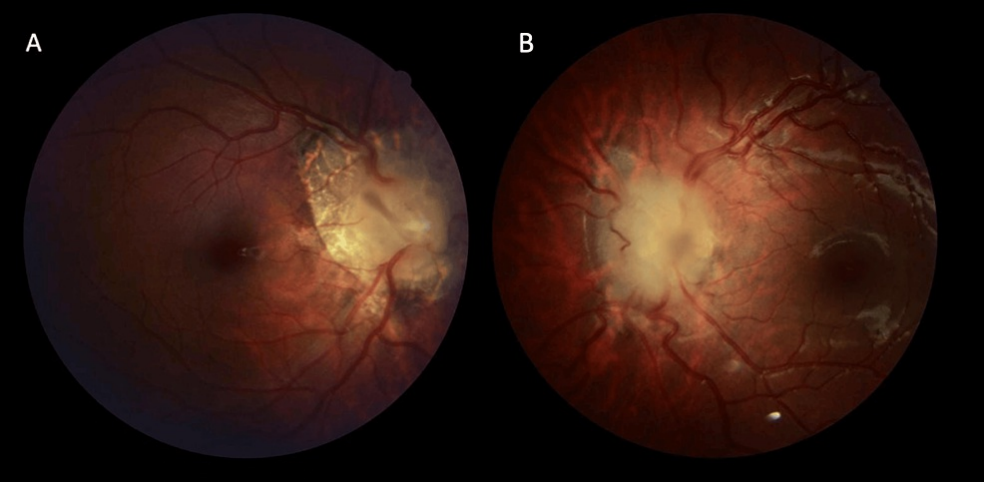
Fundus photographs of both eyes show an optic disc with blurred contours and elevated margins, from which emanates a short, brownish fibrous band. (A: right eye; B: left eye)

Fundus examination of both eyes (Figure 1) revealed congested peripapillary vessels, a markedly elevated and blurred-edged optic disc without hemorrhages or exudates, along with peripapillary chorioretinal atrophy for the right eye (Figure 1A). A short, mobile, brownish band extending from the optic disc towards the vitreous cavity was observed.

**Figure 2:**
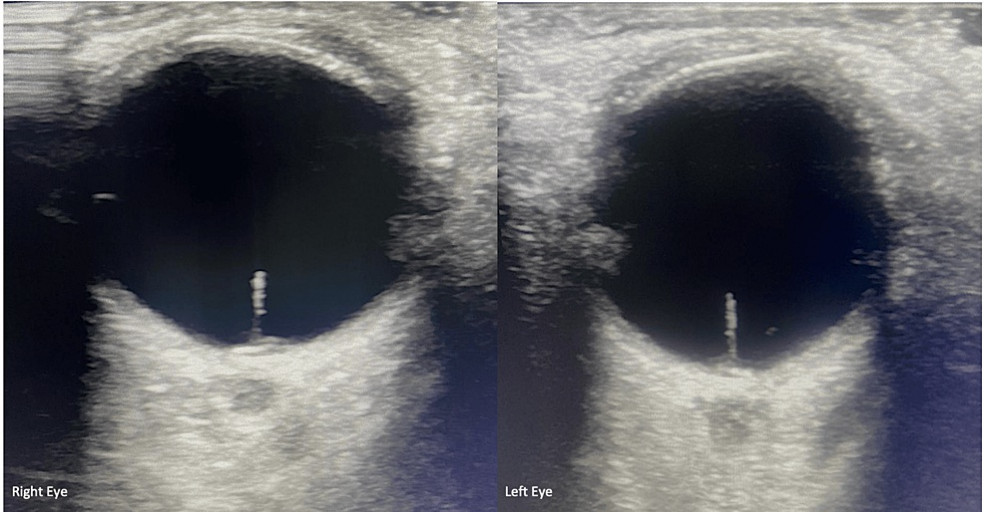
Ocular ultrasound of both eyes shows a fine hyperechoic line pulling on the optic nerve head.

Ocular ultrasound of both eyes revealed a bilaterally fine hyperechoic line emanating from the optic nerve head, causing its elevation. The axial length was measured at 19.5 mm in both eyes (Figure 2).

**Figure 3:**
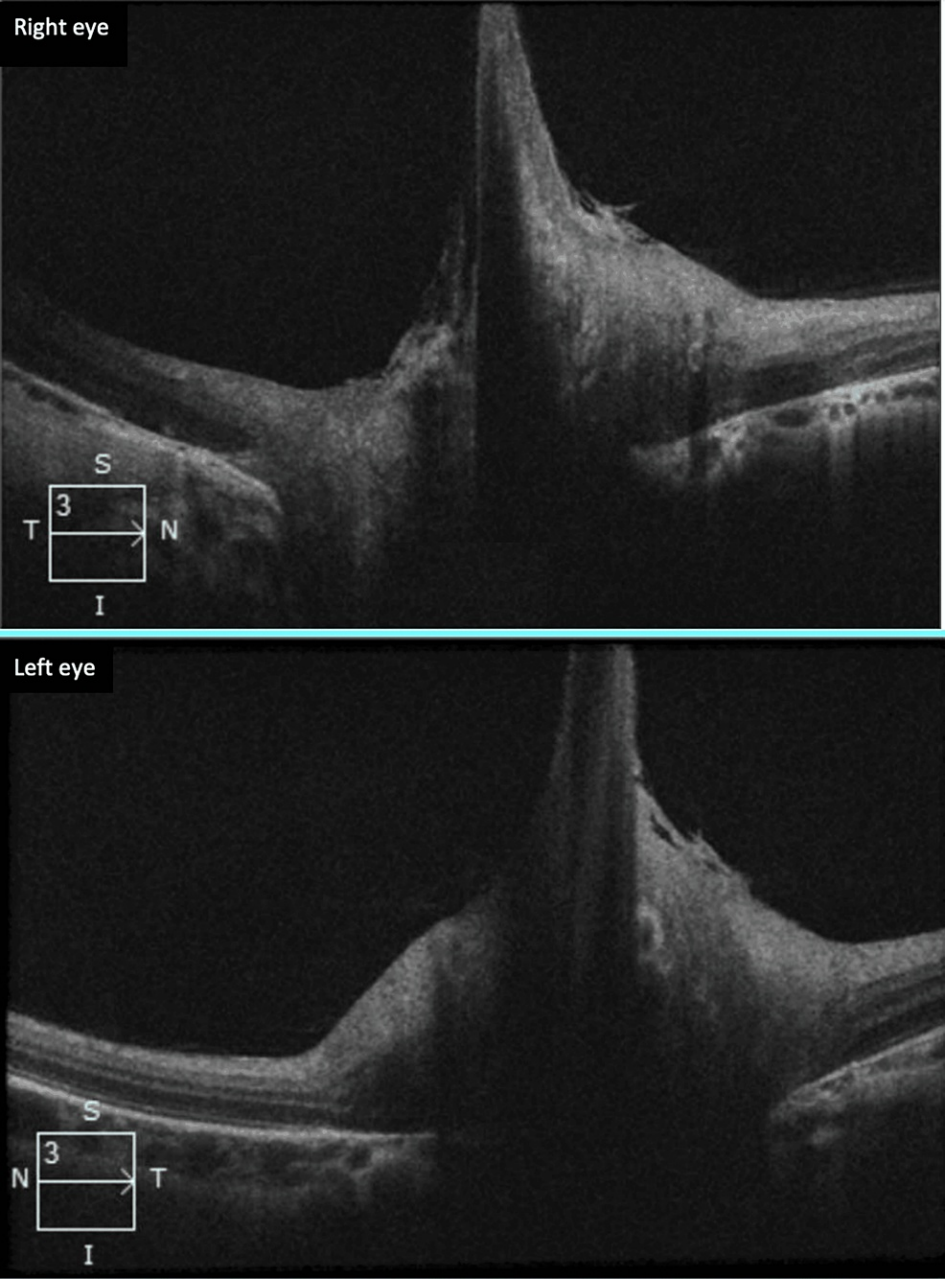
Papillary OCT of both eyes shows bilateral papillary traction on persistent fetal vascularization. OCT: optical coherence tomography

Papillary optical coherence tomography (OCT) (Figure 3) revealed papillary traction with a fibrous band pulling the optic disc towards the vitreous cavity. There was no traction on the macula and no evidence of retinoschisis.

The diagnosis of a posterior and bilateral form of persistent fetal vasculature with papillary traction was established.

Although surgical intervention could have been considered for treatment, a careful evaluation of the benefit-risk balance, given the patient's visual acuity of 20/20 and the absence of visual symptoms, led to the decision to opt for a conservative and non-surgical approach with close monitoring. The patient's condition remained stable, both anatomically and functionally, throughout the follow-up.

## Discussion

Described initially by Reese in 1945 [4], PFV, or PHPV, is a rare vitreoretinal malformation resulting from a failure of resorption of the primitive vitreous and the hyaloid vascular system during embryonic life [1]. Our case stands out due to three rare features: the form of its clinical presentation (pure posterior involvement), its bilaterality, and papillary traction giving the appearance of pseudopapilledema. 

The clinical presentation is subdivided into three forms. An anterior form, representing 25% to 35.5% of cases [5, 6], typically presents with leukocoria related to the retrolental fibrovascular membrane or posterior capsular cataract, with an unremarkable posterior pole [7]. A mixed form, the most common, represents 42% to 77% according to series [5], combining a fibrovascular membrane and a vascular band linking to the posterior capsule of the lens [7]. The posterior form, or Bergmeister papilla, the rarest, represents 12% to 22% of cases [5, 6].

In most series, hyperplastic persistence of the primitive vitreous is typically unilateral in over 90% of cases [4]. Bilateral forms account for less than 2.4% in certain series, such as the series of Pollard et al. [8]. They are generally associated with other ocular and genetic malformations [1], such as trisomy 13, trisomy 18, and Norrie disease [9]. In our case, there were no ocular or visceral malformations.

The main differential diagnosis is congenital cataract and retinoblastoma. To our knowledge, this is the first case of bilateral posterior fetal vascularization persistence complicated by papillary traction. A unilateral form of Bergmeister papilla with traction has been reported in the literature [10].

Other rare complications have been described, such as tractional retinal detachment, macular retinoschisis, and vascular thrombosis with vitreous hemorrhages [11, 12].

## Conclusions

Persistent fetal vasculature can be a devastating developmental anomaly that remains an important cause of amblyopia and visual disabilities in children. The patient presents an exceptional case with good visual acuity characterized by an atypical form of fetal vascularization, including three rare clinical features, bilaterality, posterior form, and papillary traction, which is a complication rarely described in the literature. 
